# Noninvasive Technologies for the Diagnosis of Squamous Cell Carcinoma: A Systematic Review and Meta-Analysis

**DOI:** 10.1016/j.xjidi.2024.100303

**Published:** 2024-07-20

**Authors:** Carina Nogueira Garcia, Christoph Wies, Katja Hauser, Titus J. Brinker

**Affiliations:** 1 Digital Biomarkers for Oncology Group, German Cancer Research Center (DKFZ), Heidelberg, Germany; 2 Medical Faculty, University of Heidelberg, Heidelberg, Germany

**Keywords:** Confocal microscopy, Keratinocyte tumor, Noninvasive, Optical coherence tomography, Squamous cell carcinoma

## Abstract

Early cutaneous squamous cell carcinoma (cSCC) diagnosis is essential to initiate adequate targeted treatment. Noninvasive diagnostic technologies could overcome the need of multiple biopsies and reduce tumor recurrence. To assess performance of noninvasive technologies for cSCC diagnostics, 947 relevant records were identified through a systematic literature search. Among the 15 selected studies within this systematic review, 7 were included in the meta-analysis, comprising of 1144 patients, 224 cSCC lesions, and 1729 clinical diagnoses. Overall, the sensitivity values are 92% (95% confidence interval [CI] = 86.6–96.4%) for high-frequency ultrasound, 75% (95% CI = 65.7–86.2%) for optical coherence tomography, and 63% (95% CI = 51.3–69.1%) for reflectance confocal microscopy. The overall specificity values are 88% (95% CI = 82.7–92.5%), 95% (95% CI = 92.7–97.3%), and 96% (95% CI = 94.8–97.4%), respectively. Physician’s expertise is key for high diagnostic performance of investigated devices. This can be justified by the provision of additional tissue information, which requires physician interpretation, despite insufficient standardized diagnostic criteria. Furthermore, few deep learning studies were identified. Thus, integration of deep learning into the investigated devices is a potential investigating field in cSCC diagnosis.

## Introduction

Cutaneous squamous cell carcinoma (cSCC) is the second most common form of nonmelanoma skin cancer (NMSC). Globally, North America holds the second highest incidence rate after Australia and New Zealand (Sung et al, 2021), with around 1 million surgical procedures performed to treat cSCC in 2012 in the United States alone (Rogers et al, 2015). Critically, the incidence rate is expected to increase by approximately 40% in Europe and 90% worldwide by 2040 (Ferlay et al., 2023).

Although cSCC is not often deadly, it can cause severe morbidity. The majority of cSCC is located in the head and neck area, and substantial excision is needed to treat the disease at a late stage, which can result in deformity (Pellacani and Argenziano, 2022). When surgical excision is not possible, immunotherapy and chemotherapy are possible treatment choices. It may be challenging to distinguish between different actinic keratosis (AK) grades, in situ cSCC/Bowen’s disease (BD), or even invasive squamous cell carcinoma (iSCC), but doing so is crucial for choosing the right course of treatment and avoiding multiple biopsies (Lomas et al, 2012; Ulrich et al, 2016).

Noninvasive medical devices emerged in the 1980s to improve skin cancer diagnostics in routine dermatology practice (Guida et al, 2019). Because of the advancement in dermoscopy, diagnoses have become more precise (Pellacani and Argenziano, 2022). Recently, sophisticated techniques have been introduced in clinical practice, including optical coherence tomography (OCT), reflectance confocal microscopy (RCM), and high-frequency ultrasound (HFUS). They differ in terms of resolution; penetration depth; and, therefore, clinical applicability (Marneffe et al, 2016). Although these methods surpass dermoscopy in their capabilities, they necessitate further expertise and have yet to attain widespread adoption.

Despite noninvasive technologies predominantly being developed for melanoma diagnostics, their gradual advancements have also begun to explore keratinocyte carcinomas, highlighting their potential relevance in clinical practice (Fink and Haenssle, 2017). Therefore, we conducted an extensive systematic review and meta-analysis of studies that investigate the use of noninvasive technologies in the diagnosis of cSCC. Subsequently, sensitivity and specificity of diagnostic tests were assessed, and latest advances in this area are reported.

## Results

**Figure 1:**
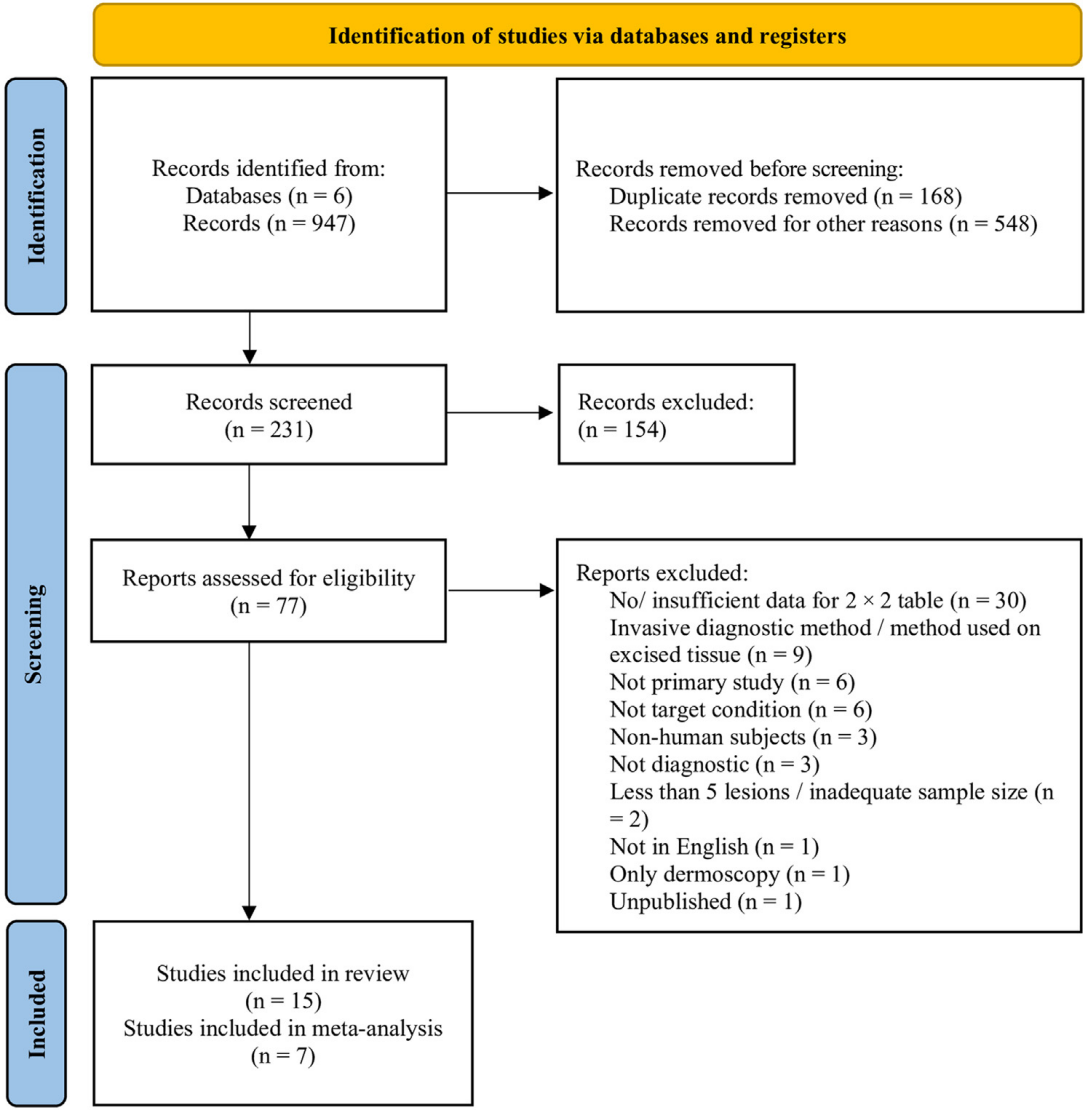
**PRISMA diagram for study selection.** Provided is the workflow of study filter and selection (Page et al, 2021). PRISMA, Preferred Reporting Items for Systematic Review and Meta-Analysis.

A total of 947 records were identified as relevant through database search. After removing 168 duplicates, 548 records were excluded during title screening. Using Covidence, 231 records were screened for abstract, and subsequently, 77 records were selected for full-text assessment. Almost half of the exclusions were based on not reported or insufficient data for 2 × 2 contingency table as shown in Figure 1.

Finally, 15 records (Boone et al, 2016, 2015; Chen et al, 2022; Díaz et al, 2019; Feng et al, 2018; Han et al, 2018; Incel et al, 2015; Jerjes et al, 2021; Longo et al, 2013; Marneffe et al, 2016; Rao et al, 2013; Rodriguez-Diaz et al, 2019; Silveira et al, 2020; Witkowski et al, 2016; Zhu et al, 2021) were included in the qualitative synthesis, of which 7 (Chen et al, 2022; Incel et al, 2015; Jerjes et al, 2021; Marneffe et al, 2016; Rao et al, 2013; Witkowski et al, 2016; Zhu et al, 2021) were selected for the quantitative synthesis.

### Studies’ characteristics

Noninvasive diagnostic technologies such as photo-acoustic, fluorescence, and hyperspectral imaging are reported in experimental or pilot studies. Most were initially developed for melanoma diagnosis and subsequently used to explore NMSC and premalignant and benign skin lesions detection and tumor surgical margin control. Full list of methods found during screening is provided in Table 1.

**Table 1:** Noninvasive Diagnostics Technologies for Skin Tumor Found on Screened Records

Method	Abbreviation	Indication/Tested on
Reflectance confocal microscopy	RCM	Human; in vivo; AK, BCC, cSCC, MM, normal skin, and benign lesions
High-frequency skin ultrasound	HFUS	Human; in vivo; AK, BCC, BD, and cSCC
Optical coherence tomography	OCT	Human; in vivo; AK, BCC, cSCC, MM, and benign lesions
Line-field optical coherence tomography	LC-OCT	Human, in vivo, MM and BCC
Dynamic optical coherence tomography	D-OCT	Human; in vivo; AK, BCC, BD, and cSCC
Single-fiber optical coherence tomography	sfOCT	Human; in vivo; tumor margins, BCC, and cSCC
Full-field optical coherence tomography	FF-OCT	Mice, in vivo, cSCC
Vibrational Optical Coherence Tomography	VOCT	Human; in vitro; AK, cSCC, BCC, and MM; normal skin in vivo
Multimodal optical coherence tomography + optical coherence tomography angiography + photoacoustic tomography	OCT/OCTA/PAT	Human, in vivo, nevus araneus
High-resolution dynamic contrast-enhanced magnetic resonance imaging	DCE MRI	Human, in vivo, invasive cSCC
Photoacoustic imaging	PAI	Human; ex vivo; BCC, cSCC, and MM
Fluorescence imaging	—	Mice; in vivo and ex vivo; BCC and cSCC
Autofluorescence imaging	—	Human, in vivo, postoperative scar tissue
Positron emission tomography	PET-CT	Human, in vivo, cSCC with subcutaneous invasiveness
Multiphoton tomography	MPT	Human, in vivo, cSCC and AK
Magnetic resonance imaging	MRI	Human, in vivo, cSCC with subcutaneous invasiveness and MM
Raman spectroscopy	—	Human; in vivo; AK, BCC, and cSCC
Optical spectroscopy	—	Human; in vivo; AK, BCC, cSCC, MM, and benign lesions
Hyperspectral imaging	HSI	Human, in vivo, and BCC and cSCC
Hybrid photoacoustic and Hyperspectral dual-modality microscopy	PAHSM	Mouse ear, in vivo, inoculated cSCC and MM cells
Electrical impedance spectroscopy	—	Human; in vivo; BCC, cSCC, MM, and benign lesions
Convolutional neural network	CNN	Human; in vivo; AK, BCC, cSCC, MM, and benign lesions
Multimodal imaging: coherent anti-stokes Raman scattering, second harmonic generation, two-photon excited fluorescence	CARS, SHG, TPEF	Human, ex vivo, BCC and SCC
Remote photoplethysmography	rPPG	Human, in vivo, cSCC and normal skin

Abbreviations: AK, actinic keratosis; BCC, basal cell carcinoma; BD, Bowen’s disease; CARS, coherent anti-stokes Raman scattering; CNN, convolutional neural network; cSCC, cutaneous squamous cell carcinoma; DCE-MRI, contrast-enhanced magnetic resonance imaging; D-OCT, dynamic optical coherence tomography; FF-OCT, full-field optical coherence tomography; HFUS, high-frequency skin ultrasound; HIS, hyperspectral imaging; LC-OCT, line-field optical coherence tomography; OCT, optical coherence tomography; OCTA, optical coherence tomography angiography; MM, malignant melanoma; MPT, multiphoton tomography; MRI, magnetic resonance imaging; PAT, photoacoustic tomography; PAHSM, photoacoustic and hyperspectral dual-modality microscopy; PAI, photoacoustic imaging; PET-CT, positron emission tomography computed tomography; RCM, reflectance confocal microscopy; rPPT, remote photoplethysmography; sfOCT, single-fiber optical coherence tomography; SHG, second harmonic generation; TPEF, two-photon excited fluorescence; VOCT, vibrational Optical Coherence Tomography.

Empty cells (—) refer to lack of information on the matter by the original study.

This review comprises 15 studies with geographical contributions from the United States (Díaz et al, 2019; Feng et al, 2018; Rao et al, 2013; Rodriguez-Diaz et al, 2019) Belgium (Boone et al, 2016, 2015; Marneffe et al, 2016), China (Chen et al, 2022; Zhu et al, 2021), Italy (Longo et al, 2013; Witkowski et al, 2016), South Korea (Han et al, 2018), Brazil (Silveira et al, 2020), Turkey (Incel et al, 2015), and the United Kingdom (Jerjes et al, 2021). Research design included diagnostic accuracy test (Boone et al, 2016; Chen et al, 2022; Han et al, 2018; Incel et al, 2015; Jerjes et al, 2021; Longo et al, 2013; Marneffe et al, 2016; Rao et al, 2013; Witkowski et al, 2016; Zhu et al, 2021), case control (Boone et al, 2015), and nonrandomized experimental studies (Díaz et al, 2019; Feng et al, 2018; Rodriguez-Diaz et al, 2019; Silveira et al, 2020). In addition, selected diagnostic methods are multimodal imaging (Díaz et al, 2019), convolutional neural network (CNN) (Han et al, 2018), Raman spectroscopy (Feng et al, 2018; Silveira et al, 2020), optical spectroscopy (Rodriguez-Diaz et al, 2019), OCT (Jerjes et al, 2021), high-definition OCT (HD-OCT) alone (Marneffe et al, 2016) and associated with an algorithm (Boone et al, 2016, 2015), RCM (Incel et al, 2015; Longo et al, 2013; Rao et al, 2013; Witkowski et al, 2016), and HFUS with Doppler (Chen et al, 2022; Zhu et al, 2021) (Table 2).

**Table 2:** Extracted Data Systematic Review

Source	Country	Sample Size	Study Design	Index Test	Model of Device or Technique	Physician Involved in Diagnoses
Marneffe et al, 2016	Belgium	71 patients; 106 images (38 AKs, 16 cSCC, and 52 normal skin)	Diagnostic test accuracy	HD-OCT	SKINTELL HD-OCT device (Agfa Healthcare Mortsel, Belgium and München, Germany)	Observer 1 (6 mo of experience with OCT); Observer 2 (1 y of experience); Observer 3 (3 y of experience)
Boone et al, 2016	Belgium	108 images (90 lesions; 18 extrinsic aged skin)	Diagnostic test accuracy	HD-OCT algorithm	SKINTELL HD-OCT device (Agfa Healthcare, Mortsel, Belgium)	Authors, no experience declared
Boone et al, 2015	Belgium	53 patients; 53 lesions (16 cSCC, 37 AK)	Case control	HD-OCT algorithm	SKINTELL HD-OCT device (Agfa Healthcare, Mortsel, Belgium)	Authors, no experience declared
Díaz et al, 2019	United States	116 patients (63 benign; 53 malignant (BCC, cSCC, MM))	Nonrandomized experiment	Multimodal imaging	P5V04A Sunny mobile visible-range mobile camera and aThermApp IR module. RaspberryPi 3B+ processing board connected to an HDMI monitor	—
Feng et al, 2018	United States	65 patients; 100 lesions (38 cSCC)	Nonrandomized experiment	Raman spectroscopy	Raman optical fiber probe integrated in an optical fiber probe–based system. An 830-nm wavelength excitation was used to minimize tissue autofluorescence. Collected signals entered a spectrograph and were imaged onto a camera integration time for each measurement was 3 s. Spectral resolution of the probe-based system is around 10 cm	—
Han et al, 2018	South Korea	>12.656 patients; >17.125 images	Diagnostic test accuracy	CNN	CNN Microsoft ResNet -152 model; Microsoft Research Asia - training with Asan dataset, MED-NODE, atlas site images (19398 imagens)	—
Incel et al, 2015	Turkey	114 patients; 122 lesions (56 BCC, 13 SK, 11 cSCC, 8 AK, 7 BD, 3 KA, 24 others)	Diagnostic test accuracy	RCM	VivaScope 3000 (Lucid, Rochester, NY)	Two independent RCM dermatology experts agreed on vascular morphology terms
Jerjes et al, 2021	United Kingdom	72 patients; 96 lesions (26 AKs, 51 BCCs, 19 cSCC)	Diagnostic test accuracy	OCT	Swept-Source Frequency-Domain Optical Coherence Tomography microscope (Michelson Diagnostics EX1301 OCT Microscope V1.0)	Reviewer previously trained in OCT interpretation
Longo et al, 2013	Italy	140 patients; 140 lesions (23 MM, 9 melanoma metastases, 28 BCC, 6 cSCC, 74 benign lesions)	Diagnostic test accuracy	RCM	VivaScope 1500 (Lucid)	Dermatologist with 5 y of experience
Rao et al, 2013	United States	334 lesions	Diagnostic test accuracy	RCM	VivaScope 1500 (CaliberID, Rochester, NY, US)	1 confocal reader, New York, NY (1 y of experience); 1 confocal reader Modena, Italy (>9 y of experience)
Rodriguez-Diaz et al, 2019	United States	787 patients; 357 lesions on test dataset	Nonrandomized experiment	Optical Spectroscopy	ESS (DermaSensor)	—
Silveira et al, 2020	Brazil	49 lesions (3 benign, 28 BCC, 7 cSCC, 11 keratosis); 25 patients	Nonrandomized experiment	Raman Spectroscopy	Dispersive Raman spectrometer, 830nm excitation, 200 mW laser power, 20s exposure time, 2 cm^-1^ resolution	—
Witkowski et al, 2016	Italy	260 lesions (114 BCC, 12 MM, 13 cSCC, 121 others)	Diagnostic test accuracy	RCM	VivaScope 1500 (Mavig, Munich, Germany)	1 reader for dermoscopy only and 1 reader for RCM
Zhu et al, 2021	China	160 lesions (54 AK, 54 BD, 52 iSCC); 160 patients	Diagnostic test accuracy	HFUS with Doppler	MyLab Class C; Esaote SpA with linear array transducer operating at 10-22MHz, or an ultrasound biomicroscopy (UBM scanner MD300SII; Meda) with a mechanically driven linear transducer operating at 50 MHz. The CDUS images were obtained using a conventional US scanner as UBM divide had no function for angiography	Experient Radiologist performed US; two radiologists analyzed images and in case of disagreements discussed with a third radiologist
Chen et al, 2022	China	133 patients (65 BCC; 68 cSCC)	Diagnostic test accuracy	HFUS with Doppler	MyLab Class C scanner (Esaote SpA, Genoa, Italy)	Radiologist >5 y of experience

Abbreviations: AK, actinic keratosis; BCC, basal cell carcinoma; BD, Bowen’s disease; CDUS, color Doppler ultrasound; CNN, convolutional neuro network; cSCC, cutaneous squamous cell carcinoma; HD-OCT, high-definition frequency ultrasound; HFUS, high-frequency ultrasound; iSCC, invasive squamous cell carcinoma; KA, keratoacanthoma; MM, malignant melanoma; OCT, optical coherence tomography; RCM, reflectance confocal microscopy; SK, seborrheic keratosis.

Some studies refer to physicians responsible for diagnoses as observers or readers. Empty cells (—) refer to lack of information on the matter by the original study.

Nevertheless, for the meta-analysis, 8 studies were excluded. Two studies (Boone et al, 2016, 2015) were excluded owing to lack of test set; only information about training set is reported. Authors were contacted; however, they failed to respond. Three studies (Díaz et al, 2019; Feng et al, 2018; Han et al, 2018) were excluded owing to paucity of data (<2 studies of each modality). Díaz et al (2019) investigated multimodal imaging using an automatic procedure for early skin-cancer screening by dynamic thermal imaging. Feng et al (2018) investigated Raman spectroscopy, and Han et al (2018) is the study selected in the review that investigated computer-assisted diagnosis (CAD) systems, more specifically, image classification of 12 skin diseases using CNN.

Subsequently, 2 studies (Rodriguez-Diaz et al, 2019; Silveira et al, 2020) were excluded owing to intense variability of defined spectra and methods, which would make a group under optical spectroscopy term strongly heterogeneous and inaccurate. Finally, Longo et al., (2013) considered the endpoint as excision or no excision instead of classifying according to the specific type of tumor and, therefore, was also excluded.

The meta-analysis therefore evaluates the sensitivity and specificity of OCT/HD-OCT, RCM, and HFUS with Doppler for cSCC diagnosis in 7 studies with 7 cohorts, corresponding to 1144 patients and 224 cSCCs. Considering that 2 studies (Marneffe et al, 2016; Rao et al, 2013) addressed evaluation by more than 1 clinician, in total, 1729 clinical diagnoses are reported. Cohort description is presented in Table 3.

**Table 3:** Meta-Analysis Cohort Description

Source	Country	Cohort Size (Patients)	Number of cSCC	Proportion Male and Female	Age Range Cohort	Age Mean Cohort	Location
Marneffe et al, 2016	Belgium	71	16	36/35	35–91	71.0 (cSCC)	19 trunk; 50 head/neck; 16 upper limbs; 21 lower limbs
Incel et al, 2015	Turkey	114	11	65/49	18–87	—	17 trunk; 93 head and neck; 12 extremities
Jerjes et al, 2021	United Kingdom	72	19	26/46	35–70	—	96 face
Rao et al, 2013	United States	334	43	—	—	—	135 trunk; 90 face; 70 upper limbs
Witkowski et al, 2016	Italy	260	13	—	—	—	—
Chen et al, 2022	China	133	68	36/32 (cSCC)	—	79.5 (cSCC)	—
Zhu et al, 2021	China	160	52	65/95	67–84 (IQR)	77 (median)	—

Abbreviations: cSCC, cutaneous squamous cell carcinoma; IQR, interquartile range.

### Quality assessment

**Figure 2:**
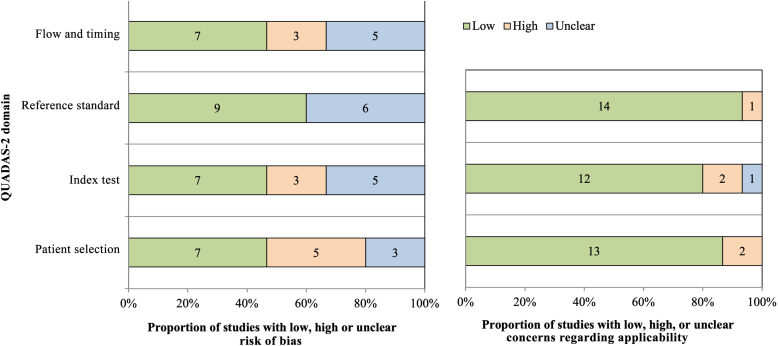
**QUADAS-2 risk of bias and applicability.** Assessment of risk of bias (left) and applicability (right) according to 4 criteria are shown: flow and timing (dependent on study design; therefore, it is not evaluated for applicability), reference standard test, index test, and patient selection. The number of studies with low, high, or unclear bias can be seen in the respective colored bars. QUADAS-2, Quality Assessment of Diagnostic Accuracy Studies 2.

A summary of the overall methodological caliber of all included study cohorts is introduced (Figure 2). According to reference standard and flow and timing, studies mostly present low or unclear risk of bias. Selective participant recruitment (5 studies), ambiguous reference test blinding (6 studies), and exclusions brought on by low picture quality and large tumor thickness are observed. Index test interpretation features mostly with low risk of bias (7 studies) because it was frequently done remotely using images, and the observer/reader was blind to any clinical information that would ordinarily be available in practice. In summary, all included studies stated blindness of examiners to clinical and histopathological information with exception of Jerjes et al (2021). Physicians also do not overlap between studies. At last, the application of the findings is not a major concern with regard to the concordance of the research question and the selected studies.

### Findings

**Figure 3:**
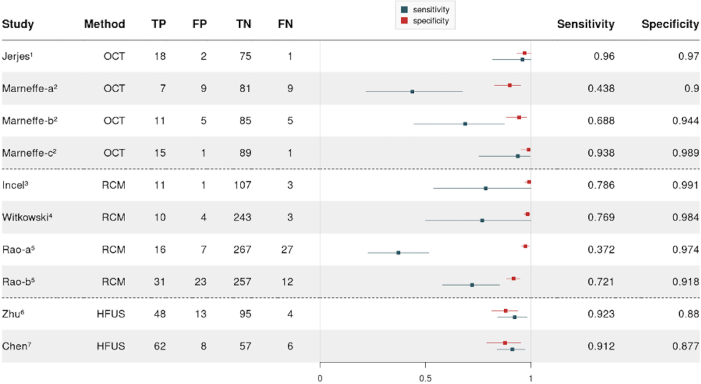
**Forest plot of sensitivity and specificity of noninvasive methods.** Metrics are shown for the 3 selected groups: OCT, RCM, and HFUS. Cohorts that were evaluated by more than 1 physician are represented by first author plus a, b, or c. CI, confidence interval; FN, false negative; FP, false positive; HFUS, high-frequency ultrasound; OCT, optical coherence tomography; RCM, reflectance confocal microscopy; TN, true negative; TP, true positive.

Studies included in the meta-analysis are clustered in 3 groups: OCT, RCM, and HFUS. These technologies are dependent on physicians to interpret the produced images. Their accuracy varies according to diagnostic criteria and physicians’ experience (Table 4). Consequently, for means of statistical analysis, cohorts that are evaluated by >1 physician (Marneffe et al, 2016; Rao et al, 2013) are separately assessed (Figure 3).

**Table 4:** Meta-Analysis Accuracy Summary

Source	Index Test	Physician Experience	Sensitivity, %	Specificity, %	95% CI for Sensitivity	95% CI for Specificity
Jerjes et al, 2021	OCT	Reviewer previously trained in OCT interpretation	96.0	97.0	81.8–100.0%	93.3–100.0%
Marneffe et al, 2016(a)	HD-OCT	6 mo	43.8	90.0	21.8–67.6%	82.7–95.1%
Marneffe et al, 2016(b)	HD-OCT	1 y	68.8	94.4	44.5–87.5%	88.4–98.0%
Marneffe et al, 2016(c)	HD-OCT	3 y	93.8	98.9	75.3–99.6%	95.4– 99.9%
Incel et al, 2015	RCM	Consensus between 2 dermatologists	78.6	99.1	53.8–100.0%	97.1–100.0%
Witkowski et al, 2016	RCM	Not declared	76.9	98.4	50.0–100.0%	96.7–99.2%
Rao et al, 2013	RCM	1 y	37.2	97.4	22.7– 51.6%	95.4–99.2%
Rao et al, 2013	RCM	9 y	72.1	91.8	58.1–85.1%	88.4–94.8%
Zhu et al, 2021	HFUS	Consensus between 2 radiologists	92.3	88.0	84.2– 98.2%	81.3–93.7%
Chen et al, 2022	HFUS	>5 y	91.2	87.7	83.8–97.1%	79.0– 95.1%

Abbreviations: CI, confidence interval; HD-OCT, high-definition optical coherence tomography; OCT, optical coherence tomography; HFUS, high-frequency ultrasound; RCM, reflectance confocal microscopy.

Two studies (Jerjes et al, 2021; Marneffe et al, 2016) report data for OCT or HD-OCT using distinct devices. Jerjes et al (2021) developed a prospective study with facial lesions, more specifically keratinocyte carcinomas and AKs. For a previously trained physician who evaluated 19 cSCCs of 72 lesions, sensitivity was 96.0% (95% confidence interval [CI] = 81.8–100.0%), and specificity was 97.0% (95% CI = 93.3–100.0%). Marneffe et al (2016) aims at the validation of a HD-OCT–based algorithm by determining its accuracy and reliability in AK/squamous cell carcinoma (SCC) classification and reported 3 physicians with different degrees of experience. The first physician reported by Marneffe et al (2016) had only 6 months of experience and achieved lowest sensitivity 43.8% (95% CI = 21.8–67.6%) and lowest specificity of 90.0% (95% CI = 82.7–95.1%). The second physician (Marneffe et al, 2016) had 1 year experience with also low sensitivity of 68.8% (95% CI = 44.5–87.5%) and high specificity of 94.4% (95% CI = 88.4–98.0%). The most experienced physician (Marneffe et al, 2016) had 3 years of experience and achieved the highest sensitivity of 93.8% (95% CI = 75.3–99.6%) and the highest specificity of 98.9% (95% CI = 95.2–99.9%).

Three studies (Incel et al, 2015; Rao et al, 2013; Witkowski et al, 2016) reported on data of RCM using VivaScope 1500 or 3000. First, Incel et al (2015) investigate vascular morphology and distribution patterns to predict NMSC diagnosis and monitor tumoral lesions. In a cohort of 114 patients, there are 13 cSCCs, and diagnosis was reached according to consensus between 2 dermatologists of no informed experience that reached 78.6% sensitivity (95% CI = 53.8–100.0%) and 99.1% specificity (95% CI = 97.1–100.0%).

Second, Witkowski et al (2016) investigate the accuracy of combined dermoscopy–RCM imaging diagnostic within a cohort of 260 nonpigmented pink lesions, including 13 cSCCs. The physician is only provided RCM images and achieves sensitivity of 76.9% (95% CI = 50.0–100.0%) and specificity of 98.4% (95% CI = 96.7–99.6%). Level of experience or previous training is not stated.

Third, Rao et al (2013) reports diagnostic accuracy of RCM images interpreted by 2 physicians investigating the biggest cohort with 340 patients and 43 cSCC in a teleconsultation setting. The first physician (Rao et al, 2013), who is less experienced, achieves lower sensitivity of 37.2% (95% CI = 22.7–51.6%); however, higher specificity of 97.4% (95% CI = 95.4–99.2%) was achieved than the second the physician (Rao et al, 2013), who has 9 years of experience. The latter achieves 72.1% sensitivity (95% CI = 58.1–85.1%) and 91.8% specificity (95% CI = 88.4–94.8%).

In regard to accuracy of HFUS with color Doppler using MyLab Class C scanner, Zhu et al (2021) focus on the ability of HFUS examination to differentiate the spectrum of cSCC on a cohort of 160 lesions, including AKs, BD, and iSCCs. Diagnosis is reached through clearly described criteria and a consensus between 2 radiologists with a sensitivity of 92.3% (95% CI = 84.2–98.2%) and specificity of 88.0% (95% CI = 81.3–93.7%). However, Chen et al (2022) explore the differentiation between high-risk basal cell carcinoma (BCC) and cSCC in a cohort of 133 patients. A 5-year experienced radiologist achieves sensitivity of 91.2% (95% CI = 83.8–97.1%) and specificity of 87.8% (95% CI = 79.0–95.1%).

**Figure 4:**
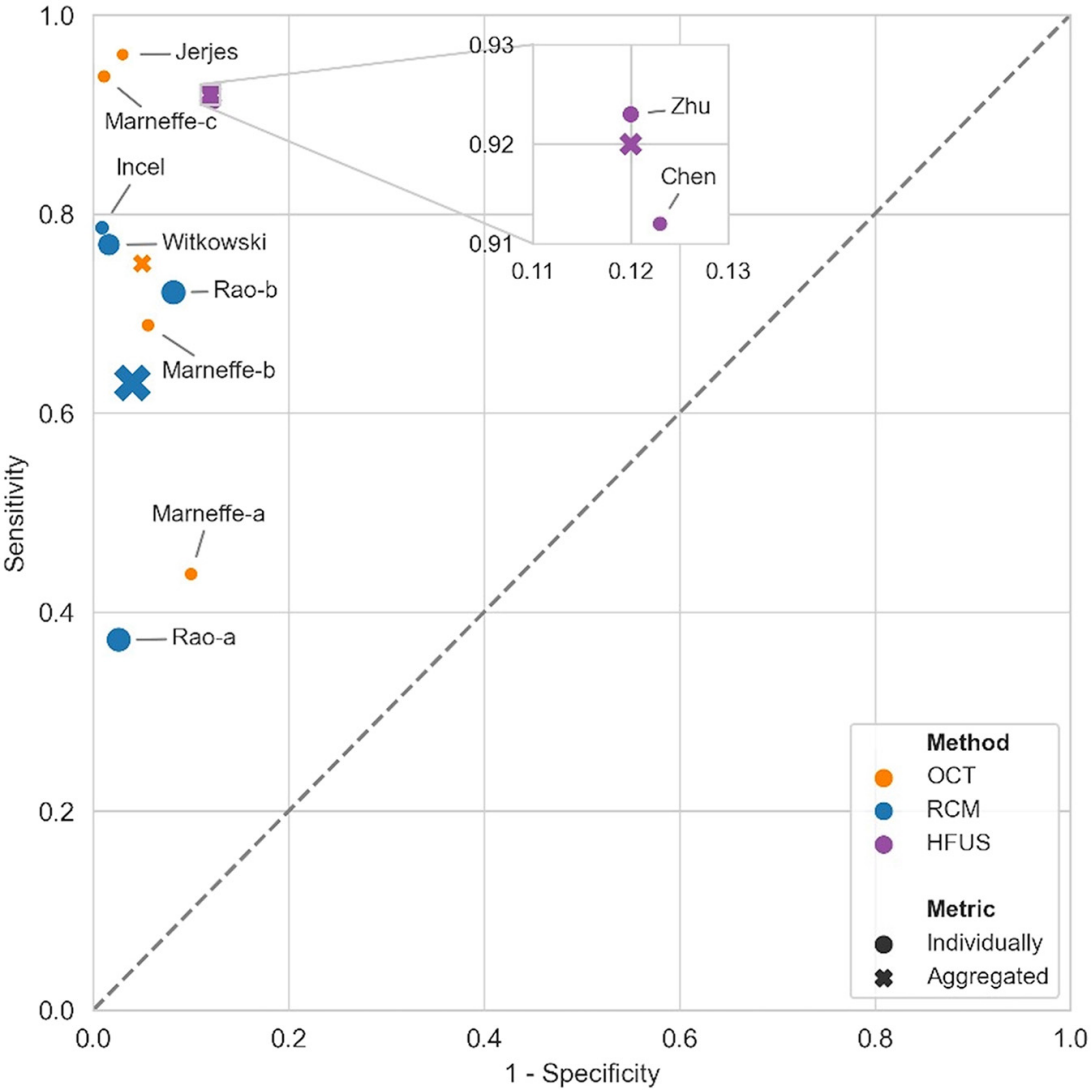
**Summary plot of overall sensitivity/specificity per method and per physician.** Individual studies/physicians are represented by dots. Overall sensitivity and specificity per method (aggregated) are represented by crosses. The size of dots and crosses varies according to sample size. HFUS, high-frequency ultrasound; OCT, optical coherence tomography; RCM, reflectance confocal microscopy.

Overall, sensitivity and specificity weighted by sample size are 75% (95% CI = 65.7–86.2%) and 95% (95% CI = 92.7–97.3%) for OCT, 63% (95% CI = 51.3–69.1%) and 96% (95% CI = 94.8–97.4%) for RCM, and 92% (95% CI = 86.6–96.4%) and 88% (95% CI = 82.7–92.5%) for HFUS with Doppler (Figure 4). Considering the first method, the total of lesions (72, 71) and cSCC images (19, 16) are similar between Jerjes et al (2021) and Marneffe et al (2016). RCM group contains the biggest cohort represented by Rao et al (2013) (360 patients and 43 cSCCs). Ultimately, HFUS studies are convergent on total of patients (133, 160) and target lesion (68, 52). Overall, accuracy is not stratified by physician experience because the latter is not reported for all 8 studies.

## Discussion

In this meta-analysis, the findings involve 3 categories of noninvasive diagnostic tools of cSCC. Studies with trained dermatologists, radiologists, or consensus in diagnosis achieve the highest sensitivity and specificity regardless of the degree of suspicion of included lesions. The majority of the studies encompass the clinical practice reality by examining malignant and benign skin lesions, with a particular focus on the diagnosis of malignant melanoma and BCC. In addition, these studies simultaneously investigate the differential diagnoses within the spectrum of cSCC, which includes AK, BD, and keratoacanthoma.

Dinnes et al (2018b) investigated the accuracy of visual inspection and in-person dermoscopy. They reported polled sensitivity and specificity of 57% (95% CI = 53–61%) and 79% (95% CI = 77–81%) for visual inspection, respectively, whereas pooled sensitivity and specificity for in-person dermoscopy was 55% (95% CI = 29–79%) and 84% (95% CI = 32–98%), respectively. However, the paucity of studies led them to state that a reliable conclusion could not be made.

In the OCT group, sensitivity and specificity range from 43.8 to 96.0% and from 90.0 to 98.9%, respectively. The highest accuracy is achieved by previously trained dermatologists. Variations of OCT for skin imaging found in this review such as high definition, full field, line field, dynamic, and single fiber provide different degrees of tissue penetration, resolution, 2-dimensional or 3-dimensional images, information on vascular structures, and tissue coordinates according to probe characteristics (Chuchvara et al, 2021). To improve quantitative evaluation, Berlin score was initially developed for BCC diagnostics. However, it still needs refinement and has not yet been validated (Wahrlich et al, 2015). The only other approach along these lines is by Marneffe et al (2016), in which applied nonvalidated diagnostic criteria did not reduce accuracy variation.

This review also revealed a higher number of studies that deployed bedside diagnostic devices, possibly because of insurance coverage, since RCM is reimbursed as an additional diagnostic method in the United States (Centers for Medicare and Medicaid Services, 2017). Consequently, this financial provision could therefore lead to wide clinical adoption.

The experience of the physicians in the RCM studies does not clearly correlate with high accuracy, possibly owing to discrepancy in sample size and absence of information on experience. Rao et al (2013) presented discordance between experience and specificity, with least experienced physician presenting higher specificity. This could in principle be explained by the different sizes of samples analyzed. However, of 334 lesions, the first physician reported by Rao et al (2013) evaluated 317, whereas the second physician (Rao et al, 2013) evaluated 323, a small discrepancy that is not enough to justify such finding. We also observed that the first physician (Rao et al, 2013) tended to diagnose more BCC instead of cSCC, a common differential diagnosis that could have contributed to a high specificity. When analyzing both metrics, the first physician (Rao et al, 2013) had a lower accuracy than the second. A previous review's findings presented limited data on the target condition but suggested sensitivity in the range of 74.0–77.0% with high specificity of 92.0–98.0% (Dinnes et al, 2018c).

HFUS with color Doppler presented convergent accuracy in both studies, probably owing to similarity in sample size and described features. On the one hand, Chen et al (2022) developed a model on the basis of ultrasound features with additional Doppler information, whereas sample selection was restricted to high-risk BCC and SCC. On the other hand, Zhu et al (2021) demonstrated accuracy on the basis of radiologist agreement. Samples included the continuum AK, BD, and iSCC (Dinnes et al, 2018a).

A comparison of technical specifications between the technologies investigated was compiled. Features include device system, wavelength, optical lateral and axial resolution, image resolution, penetration depth, field of view, and approximate imaging time (Table 5) (Agfa HealthCare NV, 2014; Cao and Tey, 2015; Chen et al, 2022; Mavig, 2018).

**Table 5:** Technical Specifications of Noninvasive Technologies

Technical Specifications	OCT	HD-OCT	RCM	HFUS with Doppler
Model	Swept-Source Frequency-Domain OCT microscope (Michelson Diagnostics EX1301 OCT Microscope V1.0)	SKINTELL HD-OCT device (Agfa Healthcare, Mortsel, Belgium)	VivaScope 3000 (Lucid, Rochester, NY), VivaScope 1500 (Lucid, Rochester, NY) VivaScope 1500 (Mavig, Munich, Germany)	MyLab Class C scanner (Esaote SpA, Genoa, Italy)
Wavelength (nm)	930–1300	1300	830	Frequency, 18–22 MHz
Optical lateral Resolution (μm)	7.5–15	3	<1.25	350
Optical axial resolution (μm)	5–10	3	<5	50
Image resolution (pixels)	256 × 256	—	1024 × 1024	—
Penetration depth (mm)	1.5–2.0	0.5–1.0	0.25–0.30	8.0
Field of view (mm)	6.0 × 6.0	1.8 × 1.5	0.50 × 0.50	12.0
Approximate time for imaging each site (min)	0.5	0.5	2 (Vivascope 3000) 10 (Vivascope 1500)	0.5
Limitations	—	High noise effect	Low penetration depth, 2D images, long capture time	Low image resolution

Abbreviations: 2D, 2-dimensional; HD-OCT, high-definition optical coherence tomography; HFUS, high-frequency ultrasound; OCT, optical coherence tomography; RCM, reflectance confocal microscopy.

Previously, Malvehy et al (2014) investigated Nevisense system performance and achieved sensitivity of 100.0% and specificity of 43.4% for cSCC diagnosis. These findings could be justified by a melanoma-focused recruitment with few differential cSCC diagnoses. This system is however trained for detecting pigmented lesions, which is not cSCC’s classical clinical presentation.

Given the recent advancements of integration of deep learning into CAD systems in melanoma diagnosis (Ferrante di Ruffano et al, 2018), we expected to find more substantial research also targeting cSCC. The only study (Han et al, 2018) found in this review that investigates CNN reports a specificity range of 82.0–90.2% and sensitivity of 74.3–80.0% in 2 different test sets. However, endpoint diagnosis was benign versus malignant.

The constraint of identified deep learning studies could reflect in the numerous challenges in its adoption (Chan et al, 2020). On one hand, compared with deployment of investigated devices within existing clinical workflows, the latter are already being used for equivocal lesions’ diagnostics. On the other hand, integration of deep learning into the investigated devices lacks substantial scientific publication in cSCC diagnostics.

To improve future studies’ design and value, we suggest the evaluation of at least 2 observers/readers with different experience levels, inclusion of visual inspection and/or dermoscopy as a control group, focus specifically on cSCC spectrum, and inclusion of patients in the field of cancerization and those immunosuppressed. A minimum of 5 target lesions is essential for an adequate 2 × 2 contingency table. Finally, the development and refinement of diagnostic criteria contributes to standardization of accuracy studies. An overview of features visualized with those technologies was performed by Danescu et al (2024) and could serve as guidance for future studies.

### Strengths and limitations

This review outlines the state of the art on cSCC noninvasive diagnostic tools. To estimate test accuracy across a variety of research populations, a thorough and repeatable investigation of methodological quality was conducted. Furthermore, a literature gap in cSCC diagnostics when concerning CAD systems was identified.

The review's key issues are first related to the inadequate reporting of primary studies—in the case of diagnostic accuracy research, this means reporting according to STARD (Standards for Reporting Diagnostic Accuracy Studies) 2015. Second, the review key issue is to the fact that not all the records were intended to be test-accuracy studies. Heterogeneous diagnostic criteria and the use of different thresholds also led to paucity of selected studies. Most of the studies are focused on pigmented lesions, leading to a limited sample size of the target condition. Intensely hyperkeratotic lesions are not considered in this review.

Third, studies with a low amount of cSCC have a lower chance for false-positive predictions; those with low frequencies have lower chances of false negatives. Therefore, we calculated pretest probability (Table 6) and found it to be highest in studies in the HFUS group. Because it positively influences the predicted metrics, the results produced should thus be interpreted with caution.

**Table 6:** Pretest Probability

Source	Cohort Size (Patients)	Lesion Total	Pretest Probability (p)	Number of cSCC	Number of Non-cSCC	BCC	BD	KA	AK/SK/SL/LP	Other
Jerjes et al, 2021	72	96	0.1979	19	77	51	0	0	26	0
Marneffe et al, 2016	71	106	0.1509	16	90	0	0	0	38	52
Marneffe et al, 2016(a)	71	106	0.1509	16	90	0	0	0	38	52
Marneffe et al, 2016(b)	71	106	0.1509	16	90	0	0	0	38	52
Marneffe et al, 2016(c)	71	106	0.1509	16	90	0	0	0	38	52
Incel et al, 2015	114	122	0.0902	11	111	56	7	3	21	24
Witkowski et al, 2016	—	260	0.05	13	247	114	0	0	25	133
Rao et al, 2013	—	334	0.1287	43	291	27	0	0	50	214
Rao et al, 2013(a)	—	317	0.1356	43	274	—	0	0	—	—
Rao et al, 2013(b)	—	323	0.1331	43	280	—	0	0	—	—
Zhu et al, 2021	160	160	0.325	52	108	0	54	0	54	0
Chen et al, 2022	133	—	0.5113	68	65	65	0	0	—	0

Abbreviations: AK, actinic keratosis; BCC, basal cell carcinoma; BD, Bowen’s disease; cSCC, cutaneous squamous cell carcinoma; KA, keratoacanthoma; LP, lichen planus; SK, seborrheic keratosis; SL, solar lentigo.

The appended letters a, b, and c correspond to different observers/readers/examiners in the same study. Rao et al (2013) reported that examiners a and b evaluated different numbers of total lesions but the same number of cSCC.

### Applicability of findings to the review question

Owing to a large array of diagnostic processes as well as in the type and performance of the equipment used and the small number of selected studies, our results may not offer sufficient data for statistical generalization. The review and meta-analysis methods are reproducible; however, the investigated studies produce metrics that might not be reproducible owing to specific examiners’ skills and particular device model. For a study comparing OCT, RCM, and HFUS within the same cohort, physicians with equivalent experience and homogeneous diagnostic criteria would be pertinent to give a general assessment about their performance.

## Conclusions

In this systematic review and meta-analysis, we found noninvasive skin cancer diagnostic tools accuracy to have a strong reliance on consensus between trained professionals as well as physician's interpretation experience with an extensive learning curve. To overcome expert dependency while improving test accuracy, we suggest further research on noninvasive cSCC detection with CAD systems and their incorporation to bedside diagnostic tools. Research targeting noninvasive tools for the detection of cSCC would substantially benefit from standardization of diagnostic criteria and adherence to reporting guidelines.

## Materials and Methods

This systematic review and meta-analysis follow PRISMA (Preferred Reporting Items for Systematic Review and Meta-Analysis) reporting guideline. To prevent data-driven analysis, it was registered with PROSPERO (International Prospective Register of Systematic Reviews) before the start of data collection.

### Search strategy and selection process

Six online scientific research databases (PubMed Central, Scopus, Web of Science, Google Scholar, IEEE Xplore, and SciELO) were searched from January to March 2023 for studies published in English since 2013 and that investigated adult human subject. The search strategy was designed in close collaboration with an experienced biomedical librarian (Supplementary Materials and Methods).

### Eligibility criteria

Studies were eligible for inclusion if they referred to a prospective or retrospective cohort of adult patients diagnosed with cSCC. The index test had to be performed in vivo before incisional or excisional biopsy was executed. Histopathology was considered as reference standard test. Studies that only reported contingency data on the basis of malignant versus benign classification were not considered (Table 7).

**Table 7:** Eligibility Criteria

Inclusion Criteria	Exclusion Criteria
Noninvasive diagnosis Cutaneous squamous cell carcinoma—skin Actinic keratosis Bowen's disease In English	Unpublished Non–peer reviewed papers Not primary study Nonhuman subjects Single case study Before 2013 Discontinued techniques in clinical practice Invasive diagnostic method Other lesions Other sites only (mucosa [lip, oral, anorectal, vulva]) 2 × 2 contingency data <5 lesions Only dermoscopy

### Data extraction and quality assessment

Two independent reviewers screened records using Covidence and extracted data using a prespecified, customized form (Table 2).

Discrepancies were resolved through mutual discussion. In case of relevant missing information, the corresponding author of the respective study was contacted. The included studies were assessed for methodological quality using STARD 2015 and the QUADAS-2 (Quality Assessment of Diagnostic Accuracy Studies 2) tool to evaluate the risk of bias and applicability (Tables 8 and 9).

**Table 8:** Risk of Bias Assessment with QUADAS-2

Study	Patient Selection	Index Test	Reference Standard	Flow and Timing
Marneffe et al, 2016	Low	Low	Unclear	Low
Boone et al, 2016	High	Low	Low	Unclear
Boone et al, 2015	High	Low	Low	Unclear
Díaz et al, 2019	Unclear	Unclear	Unclear	Unclear
Feng et al, 2018	Unclear	High	Low	Low
Han et al, 2018	High	Unclear	Low	Low
Incel et al, 2015	High	Unclear	Unclear	Low
Jerjes et al, 2021	High	High	Low	Unclear
Longo et al, 2013	Low	High	Unclear	Low
Rao et al, 2013	Low	Low	Low	Unclear
Rodriguez-Diaz et al, 2019	Low	Low	Unclear	High
Silveira et al, 2020	Low	Low	Low	Low
Witkowski et al, 2016	Unclear	Unclear	Low	High
Zhu et al, 2021	Low	Unclear	Low	High
Chen et al, 2022	Low	Low	Unclear	Low

Abbreviation: QUADAS-2, Quality Assessment of Diagnostic Accuracy Studies 2.

**Table 9:** Applicability Assessment with QUADAS-2

Study	Patient Selection	Index Test	Reference Standard
Marneffe et al, 2016	Low	Low	Low
Boone et al, 2016	Low	Low	Low
Boone et al, 2015	Low	Low	Low
Díaz et al, 2019	Low	High	High
Feng et al, 2018	Low	Low	Low
Han et al, 2018	High	Unclear	Low
Incel et al, 2015	High	Low	Low
Jerjes et al, 2021	Low	Low	Low
Longo et al, 2013	Low	Low	Low
Rao et al, 2013	Low	Low	Low
Rodriguez-Diaz et al, 2019	Low	Low	Low
Silveira et al, 2020	Low	High	Low
Witkowski et al, 2016	Low	Low	Low
Zhu et al, 2021	Low	Low	Low
Chen et al, 2022	Low	Low	Low

Abbreviation: QUADAS-2, Quality Assessment of Diagnostic Accuracy Studies 2.

### Outcomes

Our primary outcome of interest was sensitivity and specificity of diagnostic devices used for cSCC classification such as OCT, RCM, and HFUS per group as well as per physician within each group. The secondary outcome was to report currently used technologies in experimental phase or published in pilot studies.

### Statistical analysis

Statistical analysis was performed using R, version 4.1.2, using the R-package boot, version 1.3-28, as well as the base package stats. We calculated sensitivity and specificity on the basis of the 2 × 2 contingency tables reported in the studies. CIs for sensitivity and specificity were derived using the bootstrap method. For the method-wise sensitivity and specificity, we used the microaveraging approach, summing up all true positives, true negatives, false positives, and false negatives per non-nvasive method along all studies ending up in one combined sensitivity as well as specificity value per approach. The summary receiver operating characteristic curve was derived using the method suggested by Moses et al (1993). The R-package forestplot, version 3.1.1, was used to create the forest plot. The summary receiver operating characteristic plot was created using R library ggplot2, version 2.4.4.

## Ethics Statement

This study used already published data and did not directly involve human or animal subjects. The study was registered with PROSPERO (CRD42023392834)

## Data Availability Statement

No datasets were generated or analyzed during this study.

## ORCIDs

Carina Nogueira Garcia: http://orcid.org/0000-0001-9092-5952

Christoph Wies: http://orcid.org/0000-0001-7136-298X

Katja Hauser: http://orcid.org/0000-0001-9390-3505

Titus J. Brinker: http://orcid.org/0000-0002-3620-5919

## Conflict of Interest

The authors state no conflict of interest. TJB is the owner of Smart Health Heidelberg GmbH (Heidelberg, Germany), outside of the scope of the submitted work.

## Supplementary Materials and Methods

### Search terms

#### Keywords

Skin cancer/tumor or tumor/neoplasms, non-melanoma skin cancer, nmsc, squamous cell carcinoma, scc, squamous cell epithelioma, epidermoid carcinoma, epitelioma espinocelular, bowen’s disease, morbus bowen, bowenoid papulosis, keratoacanthoma, keratinocyte cancer or carcinoma, noninvasive, dermoscopy/ epiluminescence microscopy, dermatoscope, digital dermatoscope, computer assisted, computer aided, artificial intelligence, image analysis, reflectance confocal microscopy (RCM), optical coherence tomography (OCT), hyperspectral imaging, high-frequency ultrasonography (HFUS), plethysmography, Raman spectroscopy, fluorescence polarization, electrical impedance spectroscopy, and photomicrograph∗.

#### PubMed Central

(non-melanoma skin cancer [Text Word] OR non-melanoma skin cancer [Text Word] OR nmsc [Text Word] OR squamous-cell epithelioma [Text Word] OR squamous-cell epithelioma [Text Word] OR squamous-cell epitheliomas [Text Word] OR squamous-cell epitheliomas [Text Word] OR epidermoid carcinoma [Text Word] OR epidermoid carcinomas [Text Word] OR Planocellularcarcinoma [Text Word] OR Planocellular carcinomas [Text Word] OR bowen's disease [Text Word] OR morbus bowen [Text Word] OR bowenoid papulosis [Text Word] OR keratocanthoma [Text Word] OR keratinocyte cancer [Text Word] OR keratinocyte tumor [Text Word] OR keratinocyte carcinoma [Text Word] OR keratinocyte carcinomas [Text Word] OR actinic keratosis [Text Word] OR senile keratosis [Text Word] OR solar keratosis [Text Word] OR carcinoma, squamous cell [MeSH Terms] OR Skin neoplasms [MeSH Terms]) AND ((non-invasive [Text Word] OR non-invasive [Text Word]) AND (Diagnoses [Text Word] OR Diagnose [Text Word] OR Diagnostic [Text Word] OR Clinical decision making [Text Word] OR detection [Text Word] OR carcinoma, and squamous cell/diagnosis [MeSH Terms] OR skin neoplasms/diagnosis [MeSH Terms]).

#### SCOPUS

KEY (cutaneous AND squamous AND cell AND carcinoma AND diagnosis) AND PUBYEAR > 2012 AND (LIMIT-TO [LANGUAGE, English]) AND (LIMIT-TO [PUBSTAGE, final] OR LIMIT-TO [PUBSTAGE, aip]) AND (LIMIT-TO [DOCTYPE, ar] OR LIMIT-TO [DOCTYPE, re]).

#### Google Scholar

Filters: since 2013, English (cutaneous squamous cell carcinoma OR skin cancer OR actinic keratosis OR bowen’s disease AND noninvasive diagnostics).

#### IEEE Xplore

Publication range 2013 to 2023. All results. Journals. Cutaneous squamous cell carcinoma OR skin cancer OR actinic keratosis AND non-invasive diagnostics.

#### SciELO

Filters: 2013, 2014, 2015, 2016, 2017, 2018, 2019, 2020, 2021, 2022, 2023; cutaneous squamous cell carcinoma OR skin cancer OR actinic keratosis OR bowen’s disease.
